# Characterization of CD41^+^ cells in the lymph node

**DOI:** 10.3389/fimmu.2022.801945

**Published:** 2022-08-11

**Authors:** Li Dai, Mayuko Uehara, Xiaofei Li, Brenna A. LaBarre, Naima Banouni, Takaharu Ichimura, Melissa M. Lee-Sundlov, Vivek Kasinath, Jade A. Sullivan, Heyu Ni, Francesca Barone, Silvia Giannini, Baharak Bahmani, Peter T. Sage, Nikolaos A. Patsopoulos, George C. Tsokos, Jonathan S. Bromberg, Karin Hoffmeister, Liwei Jiang, Reza Abdi

**Affiliations:** ^1^ Transplantation Research Center, Renal Division, Brigham and Women’s Hospital, Harvard Medical School, Boston, MA, United States; ^2^ China Pharmaceutical University, Nanjing, China; ^3^ Systems Biology and Computer Science Program, Ann Romney Center for Neurological Diseases, Department of Neurology, Brigham & Women’s Hospital, Boston, MA, United States; ^4^ Renal Division, Brigham and Women’s Hospital, Harvard Medical School, Boston, MA, United States; ^5^ Division of Hematology, Brigham and Women’s Hospital, Harvard Medical School, Boston, MA, United States; ^6^ BloodCenter of Wisconsin, Blood Research Institute, Milwaukee, WI, United States; ^7^ Department of Laboratory Medicine and Pathobiology, and Toronto Platelet Immunobiology Group, St. Michael’s Hospital, University of Toronto, Toronto, ON, Canada; ^8^ Canadian Blood Services Centre for Innovation, Toronto, ON, Canada; ^9^ Centre for Translational Inflammation Research, University of Birmingham, Birmingham, United Kingdom; ^10^ Broad Institute of Harvard and Massachusetts Institute of Technology, Cambridge, MA, United States; ^11^ Department of Medicine, Beth Israel Deaconess Medical Center, Harvard Medical School, Boston, MA, United States; ^12^ Departments of Surgery and Microbiology and Immunology, University of Maryland School of Medicine, Baltimore, MD, United States; ^13^ Institute of Health and Medical Technology, Hefei Institutes of Physical Science, Chinese Academy of Sciences, Hefei, China

**Keywords:** CD41 ^+^ progenitor cells, lymph nodes, lymphatic remodeling, stromal cells, lymphatic

## Abstract

Lymph nodes (LNs) are the critical sites of immunity, and the stromal cells of LNs are crucial to their function. Our understanding of the stromal compartment of the LN has deepened recently with the characterization of nontraditional stromal cells. CD41 (integrin αIIb) is known to be expressed by platelets and hematolymphoid cells. We identified two distinct populations of CD41^+^Lyve1^+^ and CD41^+^Lyve1^-^ cells in the LNs. CD41^+^Lyve1^-^ cells appear in the LN mostly at the later stages of the lives of mice. We identified CD41^+^ cells in human LNs as well. We demonstrated that murine CD41^+^ cells express mesodermal markers, such as Sca-1, CD105 and CD29, but lack platelet markers. We did not observe the presence of platelets around the HEVs or within proximity to fibroblastic reticular cells of the LN. Examination of thoracic duct lymph fluid showed the presence of CD41^+^Lyve1^-^ cells, suggesting that these cells recirculate throughout the body. FTY720 reduced their trafficking to lymph fluid, suggesting that their egress is controlled by the S1P1 pathway. CD41^+^Lyve1^-^ cells of the LNs were sensitive to radiation, suggestive of their replicative nature. Single cell RNA sequencing data showed that the CD41^+^ cell population in naïve mouse LNs expressed largely stromal cell markers. Further studies are required to examine more deeply the role of CD41^+^ cells in the function of LNs.

## Introduction

Lymph nodes (LNs) are critical sites of immune homeostasis. Specialized stroma within the LNs enables the proper positioning of newly arrived T cells alongside antigens that originate from the tissues to which they are connected (1–3). Lymph flows continuously from organs into the LNs *via* afferent lymphatic ducts that open into the subcapsular sinus of the LNs, which is lined with lymphatic vessel endothelial hyaluronan receptor 1 (Lyve1)-expressing endothelial cells (4). Within the LNs, lymph moves through the cortical sinus in areas adjacent to high endothelial venules (HEVs), before it drains into the medullary sinus and exits through the efferent lymphatics. Those lymphatics eventually merge into the thoracic duct, which empties the lymph into the bloodstream (5). HEVs are extremely specialized venules found in LNs that are responsible for the entrance of naïve T cells (6, 7). Lymphatic vessels play an essential role in both the development and maintenance of the LNs (8). LNs monitor constantly the inflammatory milieu of the organs to which they are connected *via* afferent lymphatics, so they can react quickly to pathological changes occurring in these organs. Fibroblastic reticular cells (FRCs) are the prominent cells of the LN stromal compartment, but LN stromal cells (SCs) are heterogeneous, consisting of various subsets specialized to exert particular functions. Single cell genomic studies of LNs have greatly shaped our understanding of LN SCs (9, 10). FRCs are the classical LN SC, as they support the general structure of the LN, control the integrity of the microvasculature in the LN, produce chemokines responsible for the homing of T cells to the LN, and generate and maintain the extracellular matrix (ECM) of the LN (11, 12). Although FRCs are specialized in the LN with respect to their immunoregulatory function, they also share many features of other tissue stromal cells (e.g., mesenchymal stem cells), *via* their expression of mesodermal markers and replicative nature (13–15). Despite their low density in various tissues, stromal cells play a critical function in tissue injury and repair processes, and they possess the potential to serve as useful tools in the drug discovery field, as they can exert immunosuppressive effects (16, 17).

The mobilization and recruitment of progenitor cells to tissues are controlled tightly by a series of homing and adhesion molecules, including heterodimeric integrins (18). The expression of CD41 (ITGA2b or integrin αIIb) characterizes early hematopoietic stem cells (HSCs) in the embryo (19). Following the transition to definitive hematopoiesis, HSCs downregulate the expression of CD41 (20, 21). Postnatally, CD41 is considered a specific marker for the megakaryocyte lineage, and it is required for normal platelet function (22, 23). While the expression of CD41 precedes the expression of CD45 in HSCs, Ma et al (24) have reported that the acquisition of CD45 drives the differentiation of hematopoietic progenitors towards a vascular lineage. With a goal of characterizing the function of platelets in the LN, we began to examine the CD41^+^ cells in the LN. Although we were not able to detect platelets in the parenchyma of LN through CD41 expression, we identified and characterized a subpopulation of CD41^+^ cells in the LNs that expressed mesodermal markers.

## Results

### CD41+ cells are detected in proximity to the lymphatic vessels of naïve LNs

**Figure 1:**
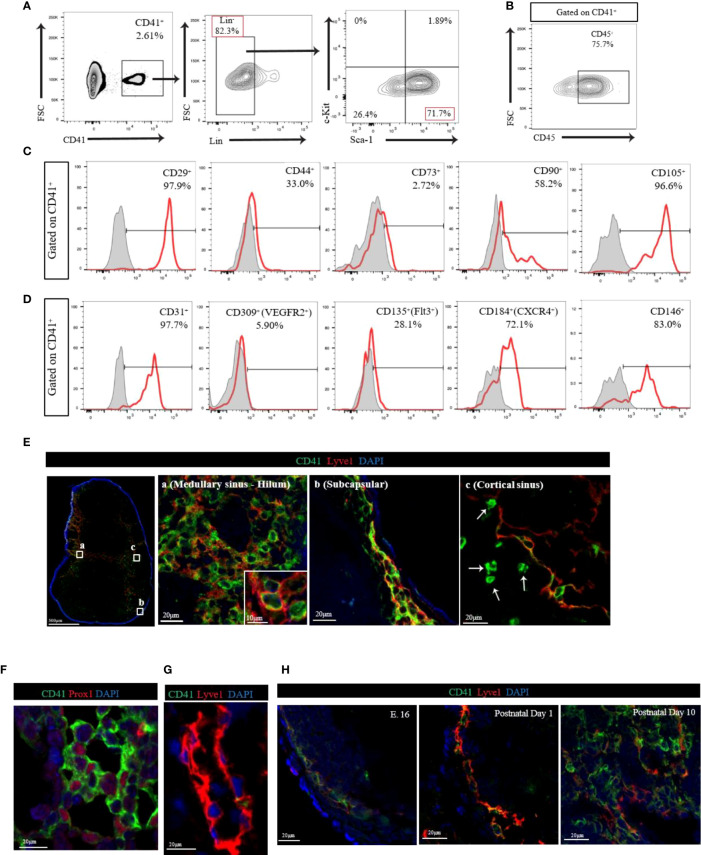
**(A)** Flow cytometry analysis of CD41^+^Lin^-^ cells from LN. The vast majority of CD41^+^ Lin^-^ cells showed positive for Sca-1, but negative for c-Kit in LN. **(B)** Flow cytometry analysis of CD41^+^ cells from LN showed high expression of CD45. **(C)** Histogram images of mesodermal stromal cell markers in LNs. High expression of stromal markers, including CD29, CD44, CD90 and CD105, was observed in CD41^+^ cells (representative image from 3 individual experiments). **(D)** Histogram images of the endothelial/progenitor cell markers in LNs showed high expression of CD31, CD184 and CD146, and moderate expression of CD135 in CD41^+^ cells (representative image from 3 individual experiments). **(E)** Confocal images in different segments of lymphatic vasculature within the naïve C57BL/6 mouse axillary LN. The vast majority of Lyve1^+^ cells co-expressed CD41 in medullary sinus-hilum (a) and subcapsular (b). CD41^+^Lyve1^-^ cells were also found in cortical sinus **(c)**. CD41 (green), Lyve1 (red) and DAPI (blue). **(F)** Co-staining of CD41 and PROX1 in naïve LN. **(G)** The thoracic duct from a naïve C57BL/6 mouse expresses Lyve1 but lacks CD41 co-expression. **(H)** IF micrograph of LNs of E.16 showed expression of CD41 in conjunction with Lyve1 expression. Both postnatal day 1 and day 10 showed also co-expression of Lyve1 and CD41 but with an increase in overall expression of Lyve1 in day 10 LNs.

We first assessed the presence of CD41^+^ cells in the peripheral LNs by flow cytometry. Flow cytometry analysis of LNs revealed that CD41^+^ cells were found mostly within larger cell size population of LNs **(** **Supplementary Figure 1A** **)**. Because hematopoietic progenitors can express CD41 (21, 25), we investigated the expression of hematopoietic stem cell (HSC) markers in CD41^+^ cells using hematopoietic lineage markers (CD3e, Ly-6G/Ly-6C, CD11b, CD45R/B220, and TER-119). We also used stem cell antigen-1 (Sca-1) and c-Kit to further delineate the mesodermal origin of these cells, and we used CD45 to examine evidence for leukocytic as well as early hematopoietic origin. We observed that 80% of CD41^+^ cells were Lin negative (CD41^+^ Lin^-^), and the vast majority of CD41^+^ Lin^-^ cells were positive for Sca-1, but negative for c-Kit (**Figure 1A**). Notably, a portion of CD41^+^ cells were positive for CD45 **(** **Figure 1B** **).** Next, we characterized CD41^+^ cells using stromal cell markers including CD29, CD44, CD73, CD90 and CD105. We observed high surface expression of CD29 (integrin β1) and CD105 (endoglin), and also the expression of CD44 (hyaluronan receptor) and CD90 (Thy-1) on CD41^+^ cells (26, 27) **(** **Figure 1C** **)**. Moreover, we assessed the expression of endothelial/progenitor cell markers, including CD31 (PECAM1), CD309 (VegfR2), CD135 (Flt3), CD184 (CXCR4), and CD146 (MCAM) in CD41^+^ cells from the LNs. High levels of CD31, CD146, and CD184 were expressed, along with moderate levels of CD135 expression **(** **Figure 1D** **)**. These data indicated that CD41^+^ cells in LNs express some of the endothelial markers. The expression of the innate lymphoid cell (ILC) marker IL-7Rα (CD127) was largely undetectable **(** **Supplementary Figure 1B** **)**. An *in vitro* colony formation assay was performed to investigate the presence of hematopoietic progenitor cells in LNs using a methylcellulose-based medium with recombinant cytokines. The BM-derived cells formed colonies, but the LN-derived cells did not grow successfully **(** **Supplementary Figure 1C** **)**.

Next, we assessed the distribution of CD41^+^ cells in naïve C57BL/6 (WT) mouse axillary LNs. We stained LNs with antibodies for CD41 and the lymphatic endothelial cell (LEC) marker Lyve1 and noticed their co-expression (**Figure 1E**). The vast majority of Lyve1^+^ cells in the medullary sinus, hilum, and subcapsular area co-expressed CD41 (**Figure 1E-a, E-b**). High-magnification of immunofluorescence (IF) images indicated that CD41 appeared to line the luminal membrane, and Lyve1 was found in the basolateral membrane of the CD41^+^ Lyve1^+^ cells **(** **Figure 1E-a**, inset). Interestingly, CD41^+^ Lyve1^-^ cells were also observed within the cortical sinus of LNs near CD41^+^ Lyve1^+^ cells **(** **Figure 1E-c**, white arrows). In addition, we stained for CD41 and Lyve1 in human LNs, and we found evidence for co-expression of CD41 and Lyve1 (**Supplementary Figure 1D**), which was consistent with the mouse LNs in **Figure 1E**. An anti-Prox1 antibody was used to support the expression of CD41 on LECs in LNs, which were characterized by expression of Prox1 in the nucleus **(** **Figure 1F** **)**. To assess the presence of CD41^+^ cells within the structure of lymphatic vessels outside the LNs, we examined the thoracic duct, which lacked CD41 expression **(** **Figure 1G** **)**. We also assessed the presence and distribution of CD41^+^ cells in the various peripheral LNs, Peyer’s patches, and thymus. The presence of CD41^+^ cells was confirmed within the lymphatic vessels of the mesenteric, pancreatic, kidney and popliteal LNs, similar to axillary LN; however, CD41^+^ cells were absent from the Peyer’s patches and thymus **(** **Supplementary Figure 2A** **)**.

Next, we examined the expression of CD41 in the LNs harvested from mouse embryos (E.16) and at postnatal days 1 and 10. Confocal images of axillary LN from E.16 embryo showed the expression of both CD41 and Lyve1 increasing over time. The axillary LN from postnatal day 1 showed the presence of CD41^+^ Lyve1^+^ cells in the subcapsular sinus, which were found to be expanded along with developed structures of lymphatics within the LN at postnatal day 10 (**Figure 1H**).

### Platelets do not co-localize with high endothelial venules (HEVs) or fibroblastic reticular cells (FRCs) in the LNs

**Figure 2:**
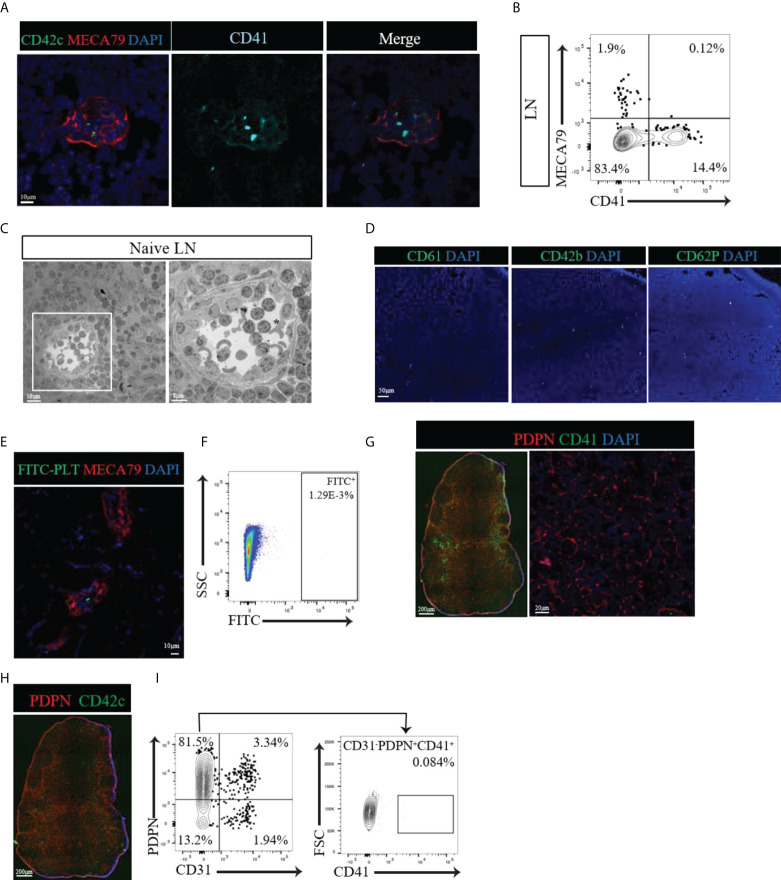
**(A)** Confocal images of naïve LNs labeled with CD41 (alcian blue), CD42c (green) and MECA79 (red). Typical CD41^+^CD42c^+^ platelets were observed only in the lumen of the HEV, not around the HEV. **(B)** Flow cytometry data showing no CD41^+^PNAd^+^ cells in naïve LNs. **(C)** Electron microscopic images of a naïve LN show platelets with granules (black asterisk) in the lumen of the HEV but not around the HEV. **(D)** A naïve LN stained for different platelet markers, such as CD61, CD42b, and CD62P, confirmed very few platelets. **(E, F)** Confocal imaging and flow cytometry assessment of LNs from mice injected with FITC-labeled platelets showed sparse populations of FITC-labeled platelets. **(G)** Immunofluorescence and confocal imaging of naïve LN showed no co-expression of CD41 and PDPN (red), CD41 (green) and DAPI (blue). **(H)** An immunofluorescence image of naïve LNs confirmed the presence of platelets in the blood vasculature and absence from the vicinity of lymphatic vessels and FRCs by staining for CD42c (green). **(I)** Flow cytometry assessment confirmed no CD41^+^ expression in the gated CD31^-^PDPN^+^ cell population.

Given that CD41 could be a postnatal marker of platelets and megakaryocytes, we investigated the distribution of platelets in the LNs through the use of several other platelet markers (CD61, CD42b, CD62P and GPIIb/IIIa). We also examined the spatial relationship of platelets to HEVs and fibroblastic reticular cells (FRCs). We observed few CD41^+^ CD42c^+^ double-positive cells with the typical platelet morphology of well-circumscribed dots within the lumen of HEVs **(** **Figure 2A** **)**. We did not observe the presence of platelets around the HEVs or within the proximity of FRCs **(** **Figure 2A** **)**. Flow cytometry also revealed the absence of a CD41^+^ MECA79^+^ population in LN **(** **Figure 2B** **)**. Electron microscopy revealed the presence of platelets (black asterisk), containing the typical ghost granules within the lumen of the HEV, but not outside the HEV wall **(** **Figure 2C** **)**. Staining of naïve LNs with several platelet markers, including CD61, CD42b, and CD62P, confirmed the presence of very few platelets within the LNs **(** **Figure 2D** **)**. To investigate the possibility that platelets pass through HEVs in large numbers, platelets isolated from peripheral blood were tagged with fluorescein (FITC) and injected into naïve wild type mice. In the LNs, the platelets were found only in the lumen of the HEVs **(** **Figure 2E** **)** and comprised a sparse population, as detected by flow cytometry **(** **Figure 2F** **)**.

Next, we investigated the location of platelets with respect to their proximity to FRCs within the interstitium of LNs, far from the location of HEVs. The FRC marker podoplanin (PDPN) is also expressed by LECs, usually along the external side of the lymphatic vasculature (Schacht et al., 2005). Within the lymphatic vasculature, CD41^+^ cells were found to be positive for PDPN **(** **Figure 2G**, left), showing that these were LECs. However, within the interstitium of the LN where FRCs reside, CD41^+^ cells did not costain with PDPN **(** **Figure 2G**, left). Higher magnification of the interstitium verified the absence of a CD41^+^ signal in proximity to FRCs **(** **Figure 2G**, right). Labeling the LNs for CD42c confirmed the presence of platelets in the blood vasculature and their absence from the lymphatic vasculature and FRC-rich interstitial compartment **(** **Figure 2H** **)**. Flow cytometry indicated no significant expression of CD41 by CD31^-^ PDPN^+^ FRCs **(** **Figure 2I** **)**. 57% of the CD31^+^ PDPN^+^ LEC population expressed CD41 (**Supplementary Figure 2B**). Together, these findings confirm the presence of CD41^+^ platelets only within the blood vasculature of naïve LNs, with no evidence of their distribution in the FRC-produced microarchitecture.

### CD41^+^ cells circulate though thoracic duct lymph (TDL)

**Figure 3:**
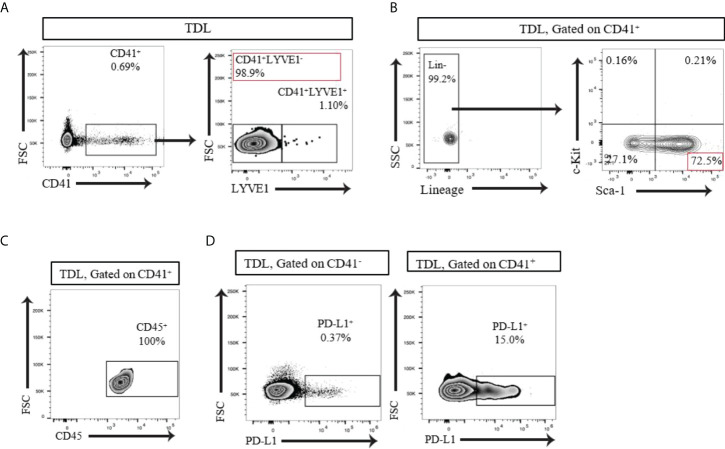
**(A)** Flow cytometry analysis of the TDL from naïve C57BL/6 mouse showed that the majority of CD41^+^ cells were negative for Lyve1. **(B)** Flow cytometry analysis of the TDL from naïve C57BL/6 mouse showed CD41^+^Lin^-^ Sca-1^+^ c-Kit^-^ cells. **(C)** All CD41^+^ cells expressed CD45 in TDL. **(D)** Flow cytometry analysis revealed that a higher percentage of CD41^+^ cells than CD41^-^ cells in the TDL from WT mice expressed PD-L1.

The LNs drains significant amount of lymph from the afferent lymphatics to the efferent lymphatics thoroughly. We examined whether CD41^+^ cells can be found in the lymph. We collected thoracic duct lymph fluid (TDL) from WT mice and detected a population of CD41^+^ cells **(** **Figure 3A** **)**. The vast majority of the CD41^+^ cells in the TDL were negative for Lyve1 (CD41^+^ Lyve1^-^) **(** **Figure 3A** **)**. Examination of HSC markers revealed that the majority of these CD41^+^ cells were Lin^-^Sca-1^+^c-Kit^-^ (LSK^-^) cells **(** **Figure 3B** **)**. Almost all of the CD41^+^ cells in TDL also expressed CD45 **(** **Figure 3C** **)**. Furthermore, CD41^+^ cells in TDL had high level of expression of PD-L1 compared to CD41^-^ cells **(** **Figure 3D** **)**.

**Figure 4:**
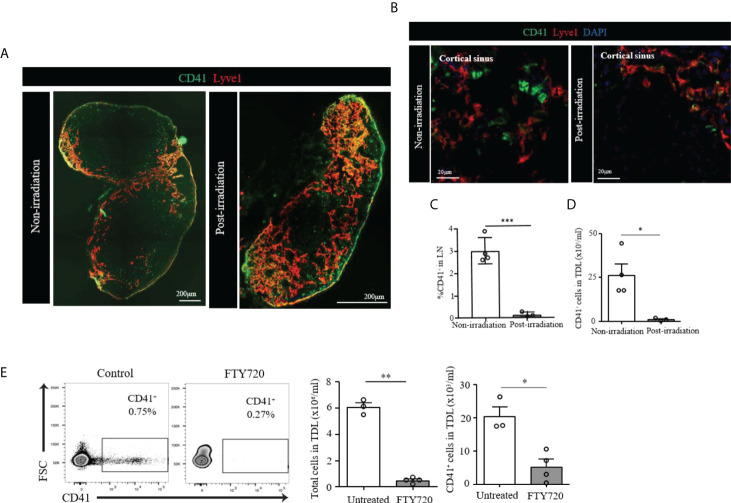
**(A)** Scanning image of whole axillary LN after irradiation showed a marked decrease of size compared to unirradiated LN. **(B)** Confocal images showed disappearance of CD41^+^Lyve1^-^ cells in the LN of post-irradiated mouse as compared to the LN of non-irradiated mouse. CD41 (green), Lyve1 (red) and DAPI (blue). **(C)** Flow cytometry assessment of LNs post-irradiation showed a marked decrease in CD41^+^ cells compared with the non-irradiation group (***p<0.001, n=3-4 mice/group). **(D)** Flow cytometry analysis showed significantly decreased CD41^+^ cell density in the TDL from an irradiated mouse compared with the TDL from a non-irradiated mouse (*p<0.05, n=3-4 mice/group). **(E)** The percentage of CD41^+^ cells were decreased in the TDL of mice treated with FTY720. The total number of cells in the TDL (**p<0.01, n=3-4 mice/group) and the absolute number of CD41^+^ cells were significantly decreased in FTY720-treated mice (*p<0.05, n=3-4 mice/group).

We then assessed the radio-sensitivity of these CD41^+^ cells. we irradiated WT mice and examined their LNs 6 days post-irradiation. The image revealed LNs of the irradiated mice were reduced in size as compared to non-irradiated LNs. CD41^+^ Lyve1^+^ cells were still present in the LN of irradiated mice **(** **Figure 4A** **)**. However, CD41^+^ Lyve1^-^ cells were largely absent in the LNs of irradiated mice, as compared to those of the non-irradiated mice **(** **Figure 4B** **)**. In corroboration with this result, flow cytometry analysis showed a significant reduction in the percentage of CD41^+^ cells in the LNs and significantly reduced number of CD41^+^ cells in TDL in comparison to the control group, which did not receive radiation **(** **Figures 4C, D** **)**. These data may suggest that CD41^+^ Lyve1^-^ cells are replicative in nature and might have originated from bone marrow.

The S1P1 pathway plays an important role in the egress of lymphocytes and HSCs from the LNs (28, 29). FTY720 is an agonist that binds S1P1 and inhibits lymphocyte egress from the LNs (29, 30). We tested FTY720 to see if the egress of CD41^+^ cells into TDL is affected. The percentage of CD41^+^ cells in the TDL from mice treated with FTY720 decreased as compared to those of the TDL from untreated naïve mice **(** **Figure 4E** **)**. In addition to the total number of cells in the TDL, the number of CD41^+^ cells were significantly decreased in FTY720-treated mice **(** **Figure 4E** **)**, suggesting that the trafficking of CD41^+^ cells to TDL is controlled by the S1P1 pathway.

### CD41^+^ cell populations expressed stromal cell markers

**Figure 5:**
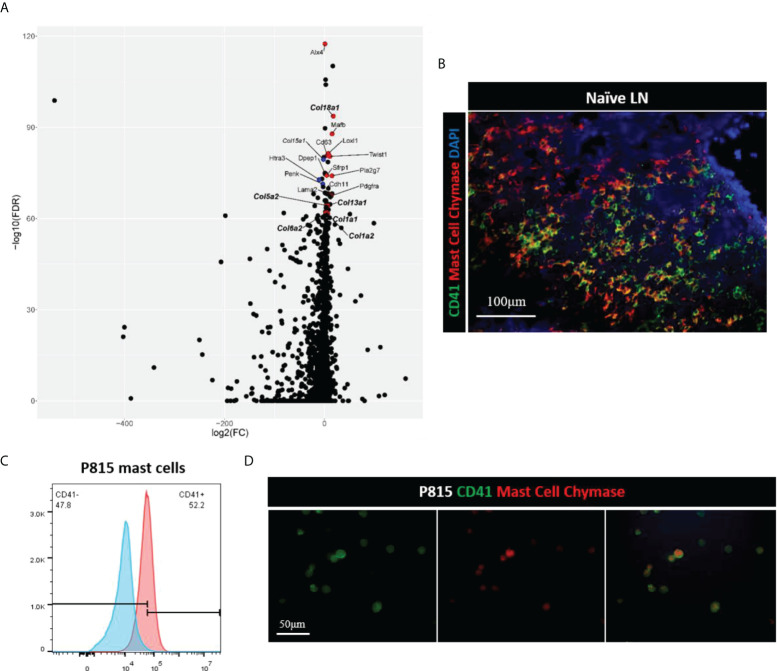
**(A)** Volcano plot of differential expression analysis of CD41^+^ cells vs. CD41^-^ cells, derived from single cell RNA-seq of naïve mouse lymph nodes. Stromal- and mast cell-associated genes are highlighted based on adjusted p-value, with the topmost gene, *Alx4*, having an adjusted p-value of 3.5 x 10^-118^, and the lowest labeled gene, *Col1a2*, having an adjusted p-value of 2.8 x 10^-62^ out of the 3,064 genes that had an adjusted p-value of < 0.05. Red dots are genes that are expressed more highly in CD41^+^ cells than CD41^-^ cells, and blue dots are those genes which are expressed less in CD41^+^ cells than CD41^-^ cells. **(B)** IF images of mouse naïve LN cells showed that mast cells (red) expressed CD41 (green). DAPI (blue). **(C, D)** Flow cytometry analysis and IF images showed mast cell line P815 (red) expressed CD41 (green).

Single cell transcriptomics has gained significant interest as a method to analyze stromal cells in the LN. These studies have helped greatly to delineate characteristics of different subsets within the LN stromal cell population (9, 10). Therefore, we performed single cell RNA sequencing of the mouse LNs to identify cell subsets that express CD41. Comparison of CD41^+^ versus CD41^-^ cells resulted in 3,064 differentially expressed genes (Bonferroni-adjusted p-value < 0.05), of which the stromal cell markers *Alx4, Mafb* and collagen genes (*col18a1, col5a2, col13a1, col1a1, col1a2, col6a2*) were enriched in the CD41^+^ populations (highlighted in red or bold) (**Figure 5A**). The mast cell marker CD63 also showed enrichment in the CD41^+^ population (**Figure 5A**), reflecting the findings of a previous study demonstrating that CD41 was expressed on mast cells (31). Therefore, we co-stained a mast cell marker (mast cell chymase) with CD41 in mouse LNs, and we found that a sizeable population of mast cells were CD41^+^ cells (**Figure 5B**). Lastly, the mast cell line P815 was found to express CD41 by flow cytometry and IF staining (**Figures 5C, D**). These data suggested that a subset of the CD41^+^ cell population could have features of stromal cells and mast cells.

## Discussion

The LN possesses a remarkably dynamic microenvironment that can support the trafficking of lymphocytes and regulate the presentation of antigens from the organ that it drains. Key to the function of LN is their stromal cells. Like any other organs, LN consists of heterogeneous SC which support its specialized function. Our understanding of LN SC has greatly increased recently with transgenic mice and newer methods of characterization of LN SC (3, 32). These studies have subsets of SC are responsible for specific function to ensure the overall function and health of LN are maintained. The FRC represents vast majority of SC differ from classical mesodermal cells with their specialized function supporting HEV, producing chemokines and building the scaffold of LN (2, 33). Even classical FRC share many features of mesodermal SC such as those of mesenchymal stem cells (34, 35). Nonetheless, LN possess classical SC cells which remain to be fully studied.

We were initially interested in examining platelet in the LN. But, we found two populations of both CD41^+^ Lyve1^+^ and CD41^+^ Lyve1^-^ cells. We detected CD41^+^ cells in virtually all LNs, although their presence was minimal to absent in Peyer’s patches and the thymus. Our data on CD41^+^ Lyve1^+^ are consistent with a report by Cordeiro et al. showing the presence of lymphatic endothelial cells of LN expressing CD41 (36). Our histological examination of human LN also supported the murine data showing the presence of CD41^+^ population in the LN.

However, here, we identified a novel population of CD41^+^ Lyve1^-^ cells which were also present in the LN. Characterization for HSC and mesodermal markers, we found that most of CD41^+^ cells in the LN expressed Sca-1 as well as other mesodermal markers such as CD105 and CD29. CD41^+^ cells were devoid of HSC markers and failed to form colony units. Interestingly, CD41^+^ cells demonstrated enrichment in vascular markers as well as CD184 (CXCR4). Our thoracic duct cannulation allowed us to show that CD41^+^ Lyve1^-^ are present in the lymph and notably express CD45 which is a pan marker for leukocytic or pan hematopoietic marker. CD41 is one of the earliest HSC markers, and its expression precedes that of CD45 (37–39). Given the CD41^+^ Lyve1^-^ cells were more sensitive to irradiation may suggest of their bone marrow origin. We then were interested in seeing whether CD41^+^ egress from LN. S1P controls the egress of lymphocytes from LNs and tissues (40, 41). FTY720 is a potent agonist of S1P receptors resulting in an impairment in lymphocyte egress (29). Notably, FTY720 treatment resulted in a significant decline in the population of CD41^+^ cells in the TDL, suggesting that S1P1 receptor engagement regulates in part the egress of CD41^+^ cells from tissues into the TDL. The egress of HSCs is also dependent on S1P, suggesting similar mechanisms of egress for both cell types (28, 42, 43). There are a number of rationales to speculate that CD41^+^ Lyve1^-^ are circulating progenitor cells which not only maintain the function of LN but may also bridge the LN microenvironment with peripheral tissues homeostatically and upon injuries. Some of the key roles of circulating progenitors are to monitor organ tissue homeostasis, repair organs, and maintain vascular integrity upon injury (44–46). The cluster of cells within the loci of aortic hematopoiesis express a mix of hematopoietic and vascular markers, including VE-cadherin, c-Kit, CD45 and CD41 (47–49). The onset of CD45 expression following CD41 expression is thought to promote a shift of the HSC to a vascular lineage. Our CD41^+^ cells lack c-Kit, indicating that they are not fully capable of hematopoietic commitment. CD45^+^/Sca-1^+^ stem cells have been previously reported to play a role in muscle repair (50, 51). A population of CD45^+^/Sca-1^+^ cells have also been described in the adventitia of aortic root in mice as well (52). CD41 and CD45 expression marks the formation of hemangioblastoma in humans (24).

Among various subsets of cells within the lymph, CD41^+^ cells harbored high levels of expression of the negative costimulatory molecule PD-L1. The latter can suggest its potent immunomodulatory function in the LN. It is also possible that following injuries to the organs and activation of LN, there is an increase in the trafficking of CD41^+^ Lyve1^-^ to LN to further support lymphatic expansion in the LN. Upon stimulation, the LN experiences significant expansion of lymphatic vessels, a process that requires a repertoire of active progenitor cells that not only maintain the lymphatic vessels in the steady state but also support the formation of new lymphatics.

These could also imply that CD41^+^ Lyve1^-^ cells could home to LN as a response to any immune activation occurring within the LN to down regulate the immune activity (expressing high PDL1) and to support lymphatic expansion by differentiating to CD41^+^ Lyve1^+^. In this regard, it is very interesting that ITGA2b expression was reported to be increased following immunization, again, highlighting the possibility that they are heterogeneous cells that can change their phenotype upon activation signals (36). There has been a significant interest in using single cell studies to further delineate LN SC subsets (9, 10). Our single cell genomic study has revealed that CD41^+^ are enriched with genes with are with the domain of mesodermal cells. It was interesting to note that at least a subpopulation of CD41 express for mast cells. This again may suggest that a subpopulation of CD41^+^ cells could be highly reactive. We have shown that mast cells could reshape the stroma of draining lymph node in a murine model of transplantation (53). Mast cells have been recognized to reside within the vicinity of the lymphatics, where they play a key role in regulating the function of lymphatics (54). Mast cells also contain vasoactive granules that can result in the expansion of lymphatics (54). Others have reported that mast cells express GPIIb integrin (CD41), which controls their adhesion (31). Collectively, these studies further highlight the need for further delineating the importance of CD41^+^ cells in the LN by using transgenic reporter and conditional knockout mice to carry out fate mapping and deletion to assess their differentiation and function.

## Methods

### Reagents

FTY720 (Cayman Chemical Company, 10006292) was purchased from Cayman, Ann Arbor, Michigan, USA. Methylcellulose-based medium (STEMCELL, MethoCult™ GF M3434) with recombinant cytokines (including EPO) was purchased for mouse cell culture from STEMCELL, Cambridge, MA, USA.

### Mice

All animal experiments and methods were performed in accordance with the relevant guidelines and regulations approved by the Institutional Animal Care and Use Committee of Brigham and Women’s Hospital, Harvard Medical School, Boston, MA (protocol number: 2016N000167/04977). C57BL/6J (WT) (#000664) and BALB/cByJ (#001026) mice were purchased from the Jackson Laboratory (Bar Harbor, ME, USA) and used at 7-8 weeks of age.

### Mouse thoracic duct lymph (TDL) collection

Thirty minutes preoperatively, 700 μL of olive oil was orally administered to mouse. The mouse was anesthetized under isoflurane and placed on its back on a heating pad controlled by a rectal sensor to maintain a constant body temperature between 36.5 and 37.5°C. The abdomen was opened, the intestines were displaced with small cotton swabs, and a self-retractor was placed in the wound. The lymphatic duct was carefully dissected from the abdominal aorta and surrounding tissue. A silastic silicone tube was tunneled subcutaneously and exteriorized through the back of the neck by means of an 18-gauge needle. The lymphatic duct was transected by micro scissors, and a silicone tube was inserted into the duct caudally and fixed by wound glue (Histoacryl). The abdominal wall was closed in two layers with a 6-0 silk running suture. The TDL was collected in an Eppendorf tube *via* the silicone tube for up to 5 hours. The TDL was centrifuged for 10 minutes at 1800 rpm, and whole cells were collected as a pellet. Where indicated, mice were treated with FTY720 (1 mg/kg) 1 day prior to TDL collection.

### Irradiation of mice

C57BL/6 mice underwent total body irradiation (9 Gy) at 85 rad/min.

### Extraction of cells from LNs and spleen

The LNs were removed and placed in 600 μl of a digestion mix containing collagenase P (0.2 mg/ml) (Roche Diagnostics, Indianapolis, IN), dispase II (0.8 mg/ml) (Sigma-Aldrich, St. Louis, MO), and DNAse I (0.1 mg/ml) (Roche Diagnostics) dissolved in Dulbecco’s phosphate-buffered saline (DPBS) (Mediatech, Inc., Manassas, VA). The LNs were placed in a water bath at 37°C for 15 minutes, followed by a round of vigorous pipetting to extract the cells. The above steps were repeated for multiple rounds of digestion until LNs were dissolved entirely and no more tissue remained in the digestion tubes. The pooled digested cells were centrifuged, resuspended in Dulbecco’s Modified Eagle Medium (DMEM) (Lonza, Walkersville, MD), and counted manually by a hemocytometer (Hausser Scientific, Horsham, PA) utilizing 0.4% Trypan blue (Thermo Fisher Scientific) to exclude dead cells.

Single-cell suspensions were extracted from spleens using a separate protocol. Each spleen was crushed over a 75-μm cell strainer (Corning Incorporated) and filtered through the strainer into a 50-mL centrifuge tube (Corning Incorporated) with 20 ml of DPBS. The cell suspension was centrifuged, the supernatant was discarded, and the pellet was resuspended with 2 ml ACK lysis buffer (Lonza) and incubated for 2 minutes at room temperature. Then, 18 ml of DPBS was added to dilute the lysis buffer, and the cell suspension was centrifuged again. The supernatant was discarded, and the pellet was resuspended in 20 ml of DPBS. A 20-μl aliquot was taken and mixed with an equal volume of 0.4% Trypan blue for cell counting *via* a hemocytometer. The cell suspension was centrifuged again, the supernatant was discarded, and the pellet was resuspended in DMEM to a concentration of 1.0 x 10^7^ cells/ml.

### Flow cytometry

Cells were plated in 96-well flat-bottom plates (Corning Incorporated, Corning, NY) for surface and intracellular transcription factor staining. All samples were washed with DPBS prior to incubation with Fixable Viability Dye eFluorTM 780 (Thermo Fisher Scientific) diluted 1:1000 in DPBS for 30 minutes at 4°C. The cells were then washed with FACS buffer (DPBS + 2% fetal bovine serum +1 mM EDTA + 0.1% sodium azide) and incubated for 25 minutes at 4°C with the following cell surface antibodies: APC anti-CD41 (MWReg30, BioLegend), PE anti-CD41 (MWReg30, BioLegend), Fluor™ 488 anti-Lyve1 (clone 223322, R&D Systems), Lineage Cocktail (17A2/RB6-8C5/RA3-6B2/Ter-119/M1/70, BioLegend), Sca-1 (D7, BioLegend), c-Kit (2B8, BioLegend), anti-CD29 (HMβ1-1, BioLegend), PE anti-CD31 (BD Biosciences), anti-CD44 (IM7, BioLegend), Pacific Blue (Pac Blue) anti-CD45 (30.F11, BioLegend), anti-CD73 (TY/11.8, BioLegend), anti-CD90.2 (30-H12, BioLegend), anti-CD105 (MJ7/18, BioLegend), anti-CD127 (Clone A7R34, BioLegend), PE anti-CD135 (Clone A2F10, Biolegend), anti-CD146 (ME-9F1, BioLegend), anti-CD184 (1.276F12, BioLegend), PerCP/Cy5.5 anti-CD309 (89B3A5, Biolegend), PE/Cy7 anti-PD-L1 (10F.9G2, Biolegend). All of the cell surface antibodies were diluted 1:500 in FACS buffer, except for PerCP/Cy5.5 anti-TER119, which was diluted 1:200. Cells were washed once with eBioscience Permeabilization Buffer (1x) (Thermo Fisher Scientific) and fixed in FACS buffer containing 1% formalin. Flow cytometry was performed using a BD FACSCantoTM II flow cytometer (BD Biosciences). Analysis of flow cytometry results was performed *via* FlowJo software (FlowJo LLC, Ashland, OR).

### Immunofluorescence

Five-micron sections of tissue were cut by cryosectioning and stained with conjugated or purified antibodies. Purified antibodies were detected using secondary antibodies. The antibodies used were FITC anti-CD41 (clone MWReg30, BioLegend), or APC anti-CD41 (clone, MWReg30, BioLegend), anti-Lyve1 (rabbit polyclonal; Abcam), anti-PROX1 (rabbit polyclonal, clone Poly19252, BioLegend), FITC anti-CD61 (#M031-1, Emfret), FITC anti-CD42b, (#M040-1, Emfret), FITC anti-CD62P (clone CLB-Thromb/6, Beckman), anti-GPIbβ (CD42c, #M050-0, Emfret), MECA79 (Santa Cruz, sc-19602), anti-PDPN (Abcam, ab11936), anti-Mast Cell Chymase (Proteintech, 18189-I-AP). The sections were visualized using the Evos FL Auto 2 microscope from Invitrogen (Thermo Fisher Scientific) for whole images and Nikon confocal microscope for high-resolution images.

### Colony formation assay

Sterile single-cell suspensions of LNs and BM from naïve C57BL/6J mice were prepared in Iscove’s Modified Dulbecco’s Medium (IMDM) containing 2% fetal bovine serum and diluted 10-fold with MethoCult™ M3434 medium (STEMCELL) to achieve a final concentration of 1x10^5^ cells/mL for LNs or 2x10^4^ cells/mL for BM. The mixtures were vortexed, placed in Petri dishes (1.1 mL/dish), and cultured in a 5% CO_2_ incubator at 37°C. After 1 week, the cells were observed, and colonies were counted under a light microscope (AmScope).

### Single cell RNA sequencing analysis

Live cells were isolated from two LNs of mice. Single cell data were generated with 10X Genomics assay by the BWH Single Cell Core. 10X sequencing data were loaded into R using the Seurat package v3.2.3 (55). Only those genes that appeared in a minimum of three cells were considered, and cells were filtered to have at least 500 but no more than 4300 genes, have at least 500 reads, and < 20% mitochondrial genes. The data were then log normalized (NormalizeData function). Next, variable features in the data were identified using the FindVariableFeatures function and default settings, which identifies 2000 variable features. The data was scaled (ScaleData function, default settings), and PCA was performed (RunPCA function, default settings). Next Harmony (R package) (56) was applied to integrate the data sets (accounting for sample run), followed by neighbor finding (FindNeighbors function, using the harmony reduction and 20 dimensions), clustering (FindClusters function, using a resolution of 0.5), and UMAP calculation (RunUMAP, also using the harmony reduction and 20 dimensions). Next, cells that expressed Itga2b were identified using the RNA count data, accounting for 204 of 3,945 evaluated cells. Using the identity of these cells, the “FindMarkers” function in Seurat was utilized to create a list of genes that are differentially expressed in the CD41^+^ expressing cells, as compared to the rest of the cells in the sample.

### Human LNs samples

Normal, fresh, iliac LNs were obtained from recipients during living donor kidney transplantation. The recipients were consented, and the study was approved by the UMSOM IRB# HP-00092098.

### Statistics

Statistical analysis of all data obtained in these experiments was performed *via* Prism 7 (GraphPad Software, Inc., La Jolla, CA) unless stated otherwise. Unpaired two-tailed Student’s t tests were performed to assess the significance of differences between two groups. The data are presented as the means ± SEM.

## Data availability statement

The original contributions presented in the study are included in the article/**Supplementary Material**. Further inquiries can be directed to the corresponding authors.

## Ethics statement

The animal study was reviewed and approved by the Institutional Animal Care and Use Committee of Brigham and Women’s Hospital, Harvard Medical School, Boston, MA (protocol number: 2016N000167/04977).

## Author contributions

LD performed the experiments, including immunohistochemistry, analyzed and interpreted the data, and drafted the manuscript. MU performed the microsurgery, analyzed and interpreted the data and drafted some of the manuscript. XL, BL, NB, TI, ML-S, JS, SG, and BB performed the experiments and analyzed the data. VK, HN, FB, PS, NP, GT, JB, and KH helped with the study design, interpreted the data, and critically revised the manuscript. LJ designed the study, interpreted the data and critically revised the manuscript. RA designed the study, interpreted the data and critically revised and finalized the manuscript. All authors contributed to the article and approved the submitted version.

## Funding

This work was supported in part by the National Institute of Allergy and Infectious Diseases of the National Institutes of Health (NIH) under award number RO1-AI170193, P01AI153003, R01-AI156084 (RA). NP was supported in part by National Multiple Sclerosis Society (grants JF-1808-32223 and RG-1707-28657).

## Conflict of interest

The authors declare that the research was conducted in the absence of any commercial or financial relationships that could be construed as a potential conflict of interest.

## Publisher’s note

All claims expressed in this article are solely those of the authors and do not necessarily represent those of their affiliated organizations, or those of the publisher, the editors and the reviewers. Any product that may be evaluated in this article, or claim that may be made by its manufacturer, is not guaranteed or endorsed by the publisher.

## Supplementary material

Supplementary Figure 1
**(A)** Gating strategy of CD41^+^ cells in LN, including size gating and single cell gating. **(B)** Flow cytometry assessment of LNs showed mostly negative expression of CD127 (IL-7Rα) in CD41^+^ cells. **(C)** A colony formation assay performed *in vitro* showed negative colony growth for LN-derived cells together with positive colony growth for BM-derived cells. **(D)** Co-staining of CD41 and Lyve-1 in human LN (isotype control staining was also included)

Supplementary Figure 2
**(A)** IF images of other secondary lymphoid organs (mesenteric LNs, pancreatic LNs, kidney LNs, popliteal LNs, Peyer’s patches, and the thymus) showed presence of CD41^+^ with the lymphatic vessels except for Peyer’s patches and the thymus. **(B)** Flow cytometry assessment confirmed that ~57% of the CD31^+^PDPN^+^ LECs cell population expressed CD41.
