# Supplementary material for: Characterization of CD41+ cells in the lymph node
Source: Front Immunol. 2022 Aug 11;13:801945. doi: 10.3389/fimmu.2022.801945 (PMC9405417; doi:10.3389/fimmu.2022.801945)

**Characterization of CD41^+^ cells in the lymph node**

**Figure legends**

**Supplementary Figure 1)**

**A)** Gating strategy of CD41^+^ cells in LN, including size gating and single cell gating. **B)** Flow cytometry assessment of LNs showed mostly negative expression of CD127 (IL-7Rα) in CD41^+^ cells. **C)** A colony formation assay performed *in vitro* showed negative colony growth for LN-derived cells together with positive colony growth for BM-derived cells. **D)** Co-staining of CD41 and Lyve-1 in human LN (isotype control staining was also included)

**Supplementary Figure 2)**

**A)** IF images of other secondary lymphoid organs (mesenteric LNs, pancreatic LNs, kidney LNs, popliteal LNs, Peyer’s patches, and the thymus) showed presence of CD41^+^ with the lymphatic vessels except for Peyer’s patches and the thymus. **B)** Flow cytometry assessment confirmed that ~57% of the CD31^+^PDPN^+^ LECs cell population expressed CD41.


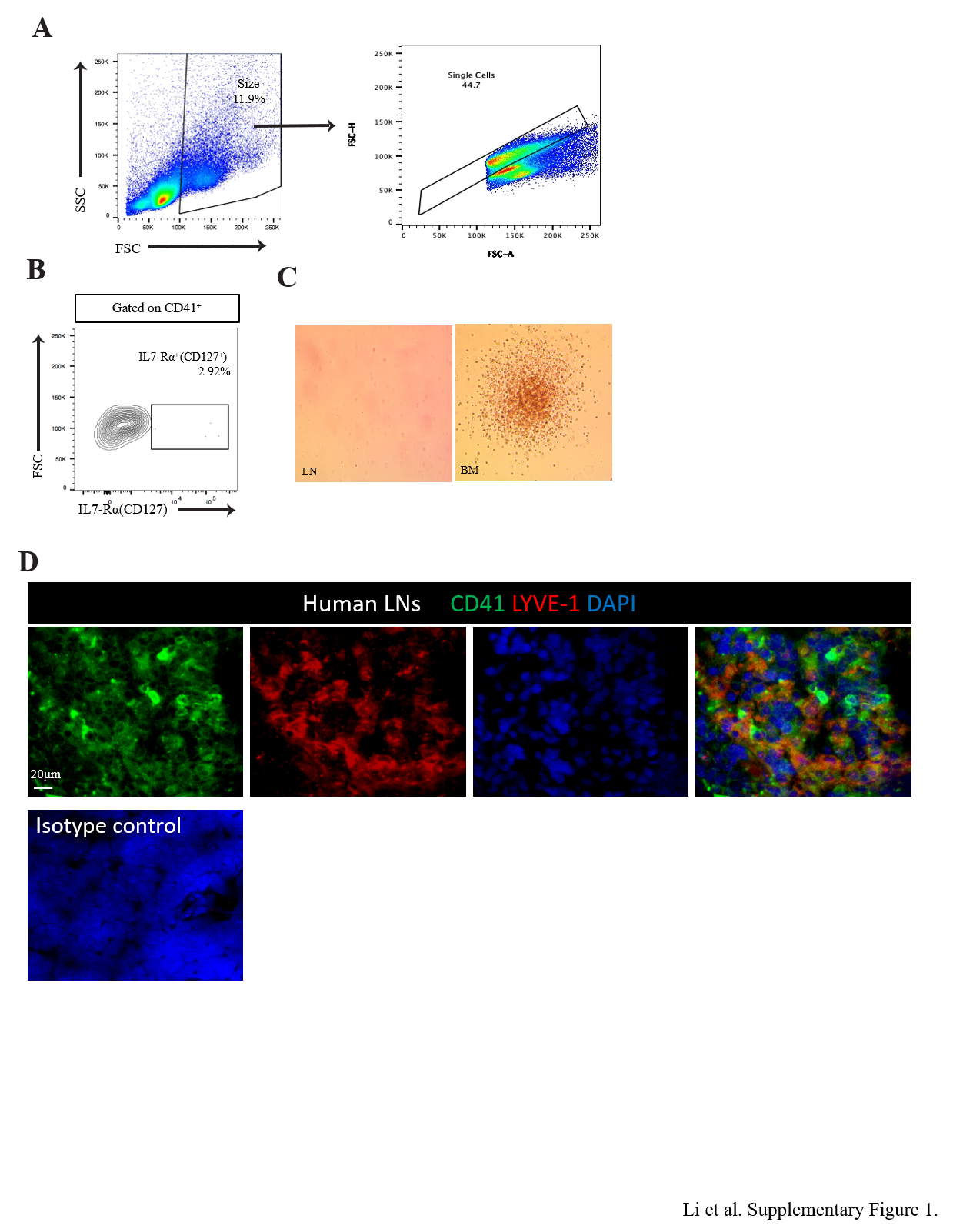


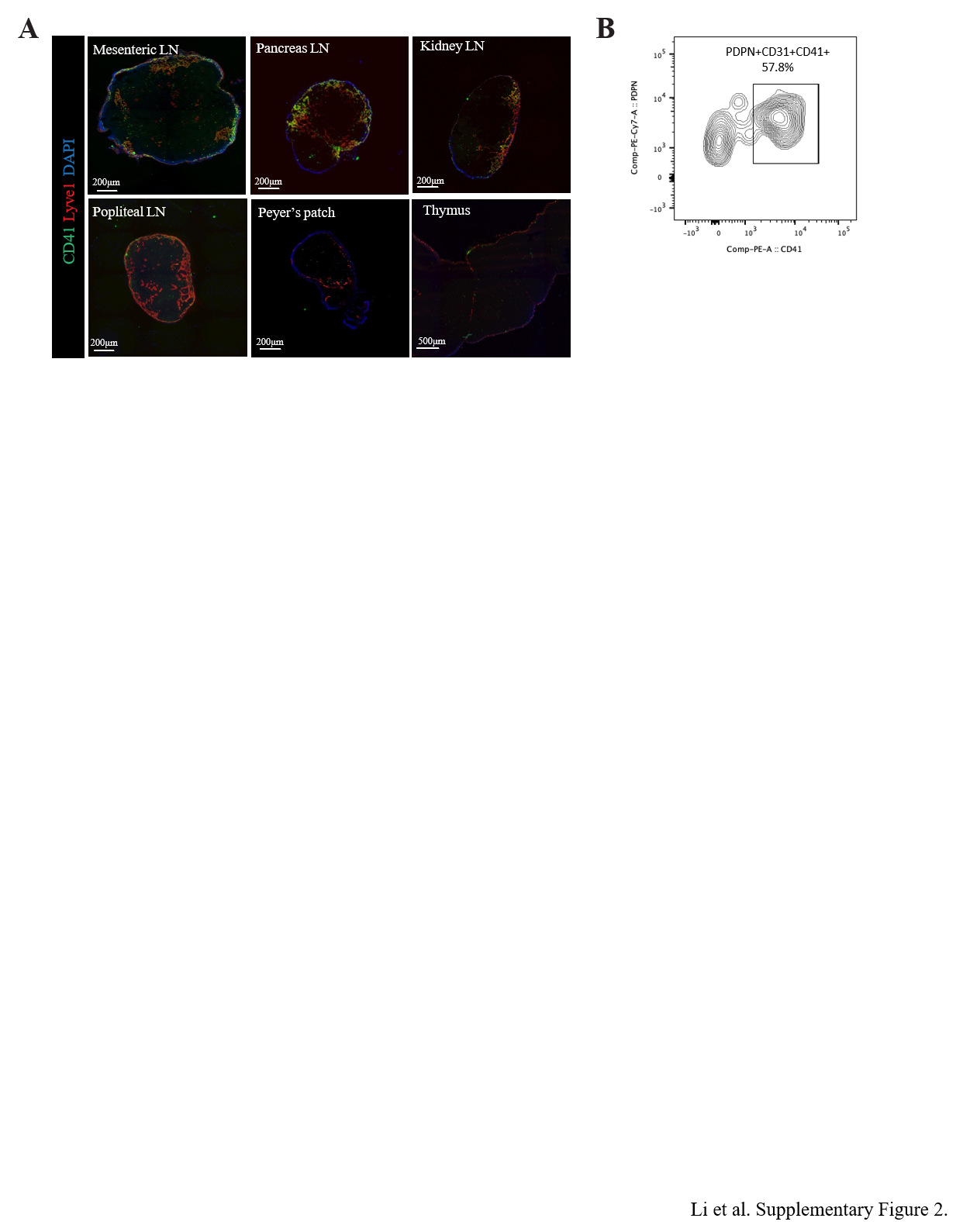

Supplement: Supplementary Figure 1 — (A) Gating strategy of CD41+ cells in LN, including size gating and single cell gating. (B) Flow cytometry assessment of LNs showed mostly negative expression of CD127 (IL-7Rα) in CD41+ cells. (C) A colony formation assay performed in vitro showed negative colony growth for LN-derived cells together with positive colony growth for BM-derived cells. (D) Co-staining of CD41 and Lyve-1 in human LN (isotype control staining was also included) [file DataSheet_1.docx]
